# RNA-seq analysis of potential lncRNAs for age-related hearing loss in a mouse model

**DOI:** 10.18632/aging.103103

**Published:** 2020-04-26

**Authors:** Tong Zhao, Xiuzhen Liu, Zehua Sun, Jinjin Zhang, Xiaolin Zhang, Chaoyun Wang, Ruishuang Geng, Tihua Zheng, Bo Li, Qing Yin Zheng

**Affiliations:** 1 Hearing and Speech Rehabilitation Institute, Binzhou Medical University, Yantai, China; 2 Clinical Medicine Laboratory, Binzhou Medical University Hospital, Binzhou, China; 3 Department of Otolaryngology-Head and Neck Surgery, Binzhou Medical University Hospital, Binzhou, China; 4 Department of Otolaryngology-Head and Neck Surgery, Case Western Reserve University, Cleveland, OH 44106 USA

**Keywords:** RNA-seq, lncRNA, age-related hearing loss, mRNA

## Abstract

Age-related hearing loss (AHL) is an important health problem in the elderly population. Its molecular mechanisms have not been fully elucidated. In this study, we analyzed the differential expression of lncRNAs and mRNAs in the cochleae of six-week-old and one-year-old C57BL/6J mice through RNA-seq analysis. We found 738 and 2033 differentially expressed lncRNAs and mRNAs, respectively, in these two groups (corrected *P* < 0.05). We focused on the intersection of known genes associated with hearing loss and differentially expressed mRNAs in RNA-seq. There are 34 mRNAs in this intersection, which include all 29 mRNAs enriched in the sensory perception of sound (GO: 0007605). We selected 11 lncRNAs that are predicted to regulate the 34 mRNAs to validate their expression levels in animal and cellular models of AHL by qRT-PCR. Among these lncRNAs, four were significantly different in both animal and cellular models of AHL, and the lncRNA NONMMUT010961.2 was the most markedly different. Knocking down lncRNA NONMMUT010961.2, we found the expression of oxidative stress and apoptosis-related gene *Ar* and hearing loss-related gene *Hgf* is significantly reduced in HEI-OC1 cells. Our results suggest that lncRNAs NONMMUT010961.2 may be associated with AHL and may thus lead to a new treatment for AHL.

## INTRODUCTION

Age-related hearing loss (AHL), also known as presbycusis, is characterized by the irreversible loss of cochlear hair cells, spiral ganglion neurons (SGNs), and stria vascularis cells (SVs) as a result of aging [1, 2]. AHL has become an important factor affecting the quality of life of the elderly. As previously reported, AHL increases the risk of depression, cognitive impairment and dementia in aged adults [3, 4]. Therefore, it is highly important to determine the molecular mechanisms of AHL and develop effective therapeutic strategies. However, the molecular mechanisms of AHL are still not well defined.

In the human genome, more than 98% of the genomic sequences do not encode protein, and only less than 2% of the regions have protein-encoding exons. For a long time, noncoding regions of the genome were thought to be junk DNA [5]. However, an increasing number of studies have revealed that noncoding RNAs (ncRNAs) regulate gene expression in different ways [6]. As a class of ncRNAs, microRNAs (miRNAs) are the most widely studied. There have been several studies on how miRNAs are related to deafness [7]. For example, in a mouse auditory cell line (HEI-OC1), miR-34 overexpression led to an increase in age-related apoptosis mediated by Bcl-2 signaling [8], SIRT1/p53 signaling [9] and autophagy activity [10]. As the largest portion of ncRNAs, long noncoding RNAs (lncRNAs), which are more than 200 nucleotides in length, are involved in many biological processes. LncRNAs are distributed in the nucleus or cytoplasm and are functional by regulating gene expression at the transcriptional, posttranscriptional, and epigenetic levels [11–13]. There are five major classes of lncRNAs: natural antisense transcripts (NATs), long intergenic noncoding RNA (lincRNA), sense overlapping RNA, sense intronic RNA, and processed transcript (a spliced and/or polyadenylated) RNA [14]. Based on lncRNA mechanisms, lncRNAs are classified into those that act in *cis* or in *trans*. *Cis*-acting lncRNAs refer to lncRNAs influencing the expression of nearby genes (the distance between lncRNA and mRNA is less than 300 kb). *Trans*-acting lncRNAs could leave the site of transfection and operate at distant sites (the distance between lncRNAs and mRNAs is more than 300 kb) [15, 16]. An increasing number of lncRNAs have been reported to be associated with the induction of cell apoptosis, tumorigenesis, and neurological disorders [6, 17]. It has also been proposed that lncRNAs contribute to neural aging by regulating pathological protein and ion channels of neuronal cells [18]. However, as an age-related neurological disorder, AHL has not been reported to have a relationship with lncRNAs. In this study, we explored the role of lncRNAs in AHL.

In this study, we first compared the expression levels of mRNAs and lncRNAs in the cochleae of six-week-old and one-year-old mice through high-throughput RNA sequencing. Then, we identified and verified the key lncRNAs in AHL and preliminarily validated the ceRNA mechanisms of the lncRNAs and mRNAs.

## RESULTS

### C57BL/6J mice were used as the animal model of AHL

**Figure 1:**
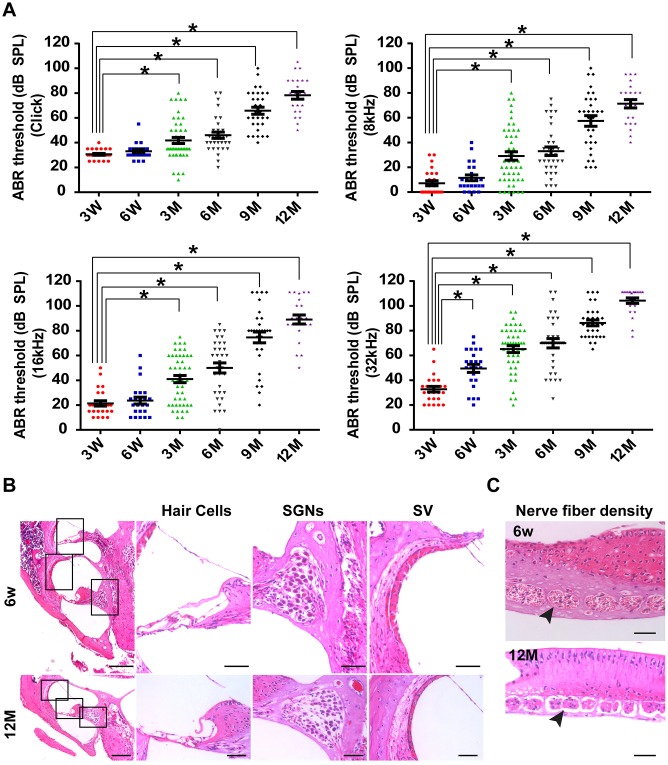
**C57BL/6J mice as the animal model of AHL.** (**A**) Elevation in auditory brainstem response thresholds was observed in aging C57BL/6J mice (n = 45) at click, 8, 16, and 32 kHz. (**B**) H&E histology shows the development of cochlear structures and pathology as age increased. *P < 0.05 compared to control; scale bars of 200 μm (left panel) and 50 μm (right panel). (**C**) Nerve fiber density in habenular openings at mid-basal turn in six-week-old and one-year-old B6 mice (scale bar = 50 μm).

The C57BL/6J mouse strain is one of the most widely used models for the study of aging and age-associated diseases [2]. By tracking the time course of the ABR threshold in C57BL/6J mice (n = 45) at click, 8 kHz, 16 kHz, and 32 kHz from three weeks old to one year old, we found that hearing thresholds progressively increased with age in C57BL/6J mice (Figure 1A). Aged C57BL/6J mice display increased hearing thresholds and large intragroup differences (Figure 1A). The ABR thresholds from three months of age onward were significantly higher than those of three-week-old mice at click, 8 kHz, and 16 kHz (*P <* 0.05). Moreover, significant differences were observed in ABR thresholds between three-week-old and six-week-old mice at 32 kHz (*P <* 0.05).

To determine the reason for the increased ABR thresholds, a cochlear histological study was performed using H&E staining. It was previously reported that both IHC and OHC were lost by one year of age in C57BL/6J mice [1, 2, 19]. In this study, our results were consistent with previous studies (Figure 1B). In addition, we found that the width of the SVs became significantly narrower from six weeks old to one year old (Figure 1B). Moreover, SGNs were clearly less abundant in one-year-old mice than in six-week-old mice (Figure 1B). Remarkably, nerve fiber densities in habenular openings of six-week-old mice were different from the densities of one-year-old mice (Figure 1C). Nerve fibers were sparser and fewer in one-year-old mice than in six-week-old mice. In general, by 12 months of age, hair cells and SGNs were lost, and the density of the SVs decreased in C57BL/6J mice. The pathology of hair cells, SGNs and SVs in one-year-old C57BL/6J mice was similar to the pathology of presbyacusis in humans [20]. Therefore, one-year-old C57BL/6J mice were chosen as the animal model of AHL in this study.

### Differential expression of lncRNAs and mRNAs between six-week-old (6w) and one-year-old (12M) mice

**Figure 2:**
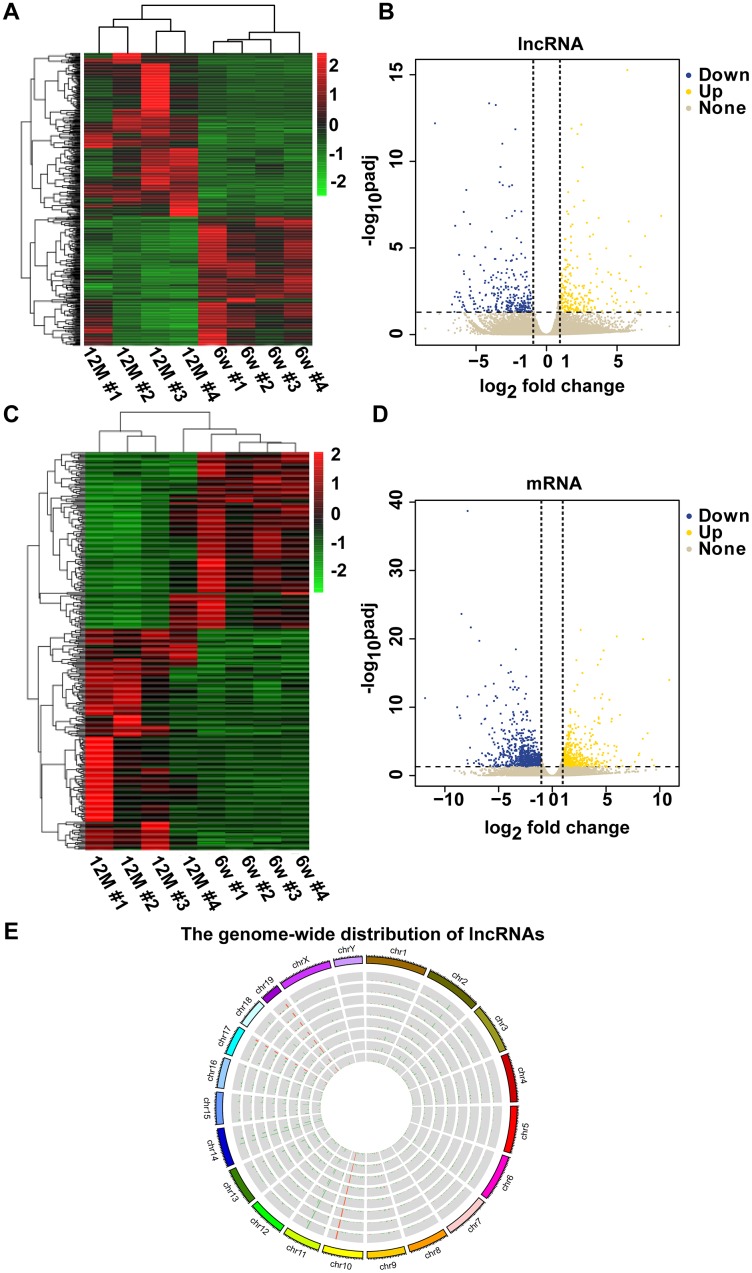
**Clustering analysis of differentially expressed lncRNAs and mRNAs in a pair of six-week-old and one-year-old mice.** The heat maps show the expression levels of differentially expressed lncRNAs (**A**) and mRNAs (**C**) between six-week-old and one-year-old mice. Items in red represent upregulated lncRNAs or mRNAs, and green items represent downregulated lncRNAs or mRNAs. Each row exhibited a lncRNA or mRNA, and each column exhibited a sample. The gradient color barcode at the top right indicates the log_2_ FPKM. Scatter plots of all expressed lncRNAs (**B**) and mRNAs (**D**) in six-week-old versus one-year-old mice. The X-axis presents the log_2_ fold change of lncRNA or mRNA expression. Yellow indicates upregulated lncRNAs or mRNAs, blue indicates downregulated lncRNAs or mRNAs, and gray indicates nonregulated lncRNAs or mRNAs. The vertical dotted lines indicate |log_2_ fold change| = 1. (**E**) Circular representation of the genome-wide distribution of the expression of detected lncRNAs by RNA sequencing. Each single circle represents a sample.

To determine the mechanism of RNAs in mouse AHL, we analyzed lncRNA and mRNA expression in cochleae of six-week-old and one-year-old C57BL/6J mice by RNA-seq. Screening was conducted based on a corrected *P*-value less than or equal to 0.05, a |log_2_ fold change| > 1. Compared with six-week-old mice groups, 289 lncRNAs were upregulated, and 449 lncRNAs were downregulated, in one-year-old mice groups (Figure 2A, 2B). In addition, compared with six-week-old mice groups, there were 683 upregulated mRNAs and 1,350 downregulated mRNAs in one-year-old mice groups (Figures 2C, 2D). Mouse genome mapping with sequenced fragments showed global changes in lncRNAs in AHL (Figure 2E). These results indicate that lncRNA and mRNA dysregulations is involved in the development of AHL in C57BL/6J mice.

### Hearing loss-related differentially expressed mRNAs

**Figure 3:**
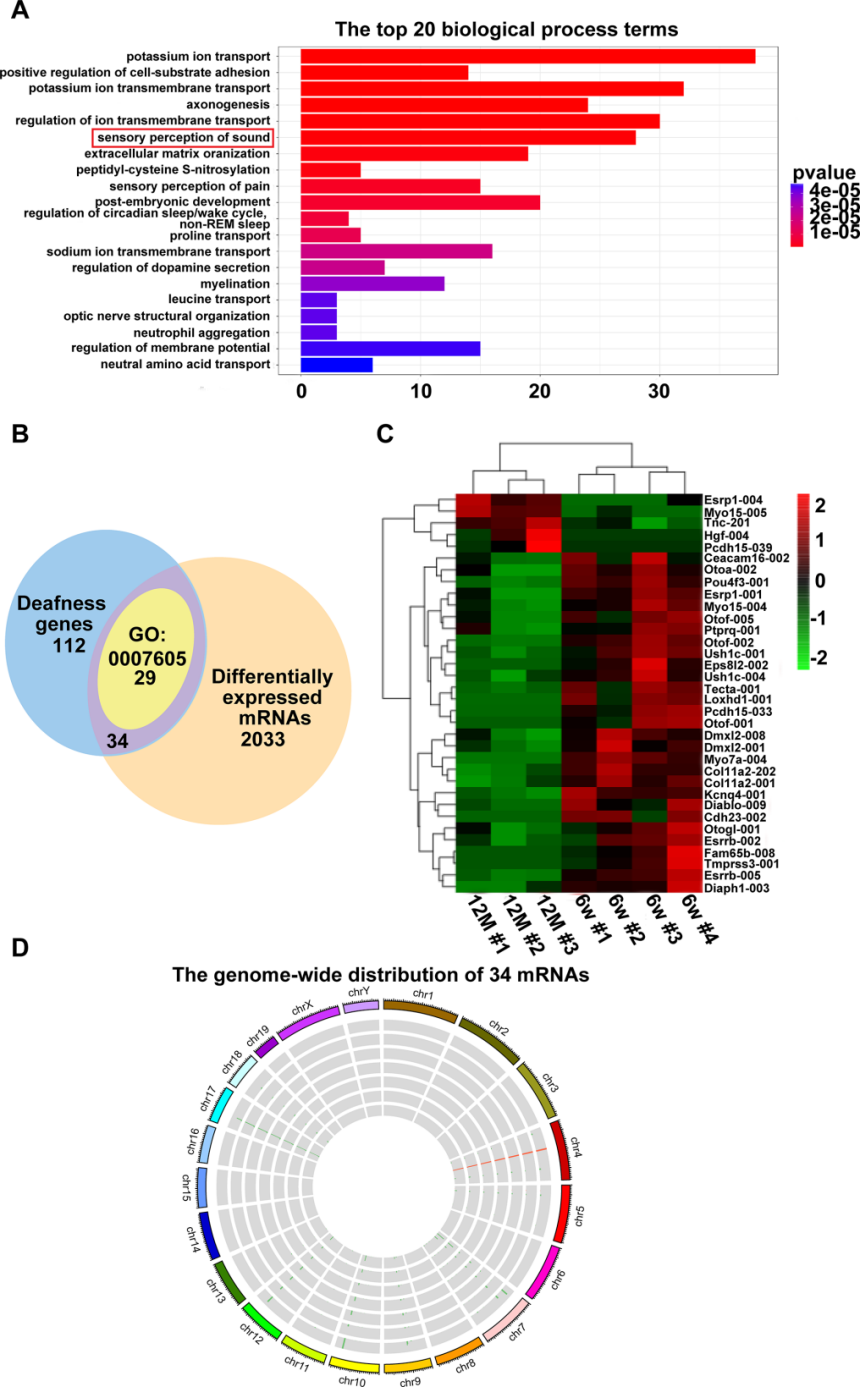
**Hearing loss-related differentially expressed mRNAs.** (**A**) Enriched biological process of six-week-old versus one-year-old mice. The x-axis indicates the number of differentially expressed mRNAs, and the y-axis indicates the top 20 biological process terms. (**B**) Venn diagram showing the intersection of popular genes associated with hearing loss, differentially expressed genes in RNA-seq, and genes enriched in sensory perception of sound (GO:0007605). (**C**) Hierarchical clustering of the 34 mRNAs. Green to red indicate the low-to-high expression levels. The gradient color barcode at the top right indicates the log_2_ FPKM. (**D**) Thirty-four mRNAs mapped to a chromosome. Each single circle represents a sample.

To demonstrate which mRNAs are involved in AHL, we first analyzed the top 20 biological processes of GO terms (Figure 3A, corrected *P <* 0.05). We found that twenty-nine mRNAs were enriched in sensory perception of sound (GO:0007605). Then, a Venn diagram was constructed with the following two groups of mRNAs. One hundred twelve genes associated with deafness were sorted from https://hereditaryhearingloss.org/updates as group 1 mRNAs. Two thousand thirty-three differentially expressed mRNAs between six-week-old and one-year-old mice in the RNA-seq data were collected as mRNA group 2 (corrected *P <* 0.05). As shown in Figure 3B, there are 34 mRNAs that are shared by the two groups, which include all 29 mRNAs enriched in sensory perception of sound. The heat maps demonstrate that the 34 mRNAs were significantly dysregulated in the one-year-old group compared with the six-week-old group (Figure 3C). The 34 differentially expressed deafness mRNAs were mapped to the mouse genome (Figure 3D). These results indicate that the 34 mRNAs involved in deafness were highly related to the development of AHL in C57BL/6J mice.

### Cellular model of AHL was established by H_2_O_2_ exposure

**Figure 4:**
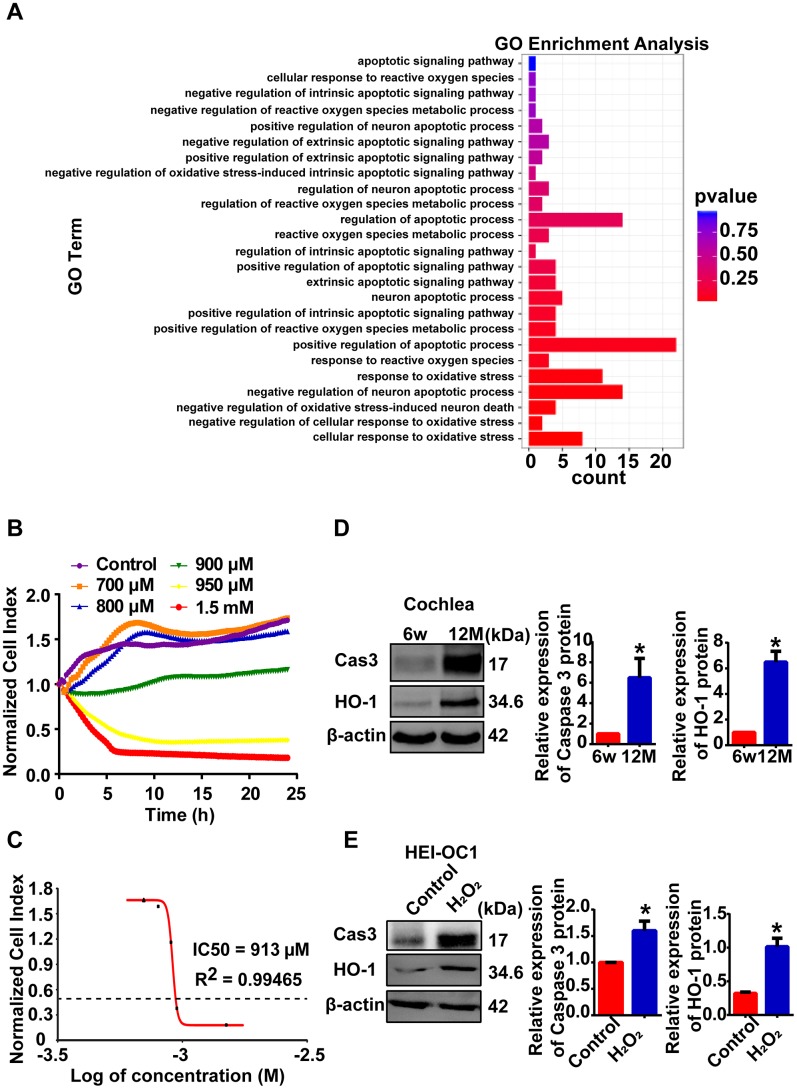
**Cellular model of AHL was established by H_2_O_2_ exposure.** (**A**) GO-BP analysis showed that oxidative stress and apoptotic processes stimulated the process of AHL. (**B**) Percent HEI-OC1 cell survival after exposure to increasing concentrations of H_2_O_2_ for 24 h, and the IC50 (half maximal inhibitory concentration) of H_2_O_2_ was approximately 920 μM (**C**). Cell viability was detected by RTCA. Cleaved caspase 3 and HO-1 expression in mouse (**D**) and cellular (**E**) models of AHL, as determined by Western blotting. *P < 0.05 compared to the control.

Reactive oxygen species (ROS) are considered the primary cause of AHL [2]. As previously reported, ninety-two genes were enriched in the oxidative stress and apoptotic processes according to GO-BP analysis in our RNA-seq data (Figure 4A). H_2_O_2_ exposure is a common way to induce oxidative stress in HEI-OC1 cells [9, 21, 22]. In this study, we established a cellular model of AHL in HEI-OC1 cells by H_2_O_2_ exposure for further lncRNA mechanism analysis. First, HEI-OC1 cells were exposed to H_2_O_2,_ and cell growth and viability were measured using a real-time cell analyzer. Exposure of HEI-OC1 cells to different concentrations of H_2_O_2_ for 24 h resulted in a remarkable dose-dependent reduction in cell growth and viability (Figure 4B). The calculated half maximal inhibitory concentration (IC50) of H_2_O_2_ is approximately 913 μM (Figure 4C). Then, we examined oxidative stress-related and apoptosis-associated protein expression in mouse cochlear tissues (Figure 4D) and HEI-OC1 cells (Figure 4E). Western blot analysis showed that heme oxygenase-1 (HO-1) and cleaved caspase 3 were significantly increased in both aged mice and cellular models of AHL compared with the control group (Figure 4D, 4E). These findings suggest that we can use HEI-OC1 cells as a cell model of AHL when exposed to H_2_O_2_ at a concentration of 920 μM for 24 h.

### mRNA-lncRNA coexpression network

**Figure 5:**
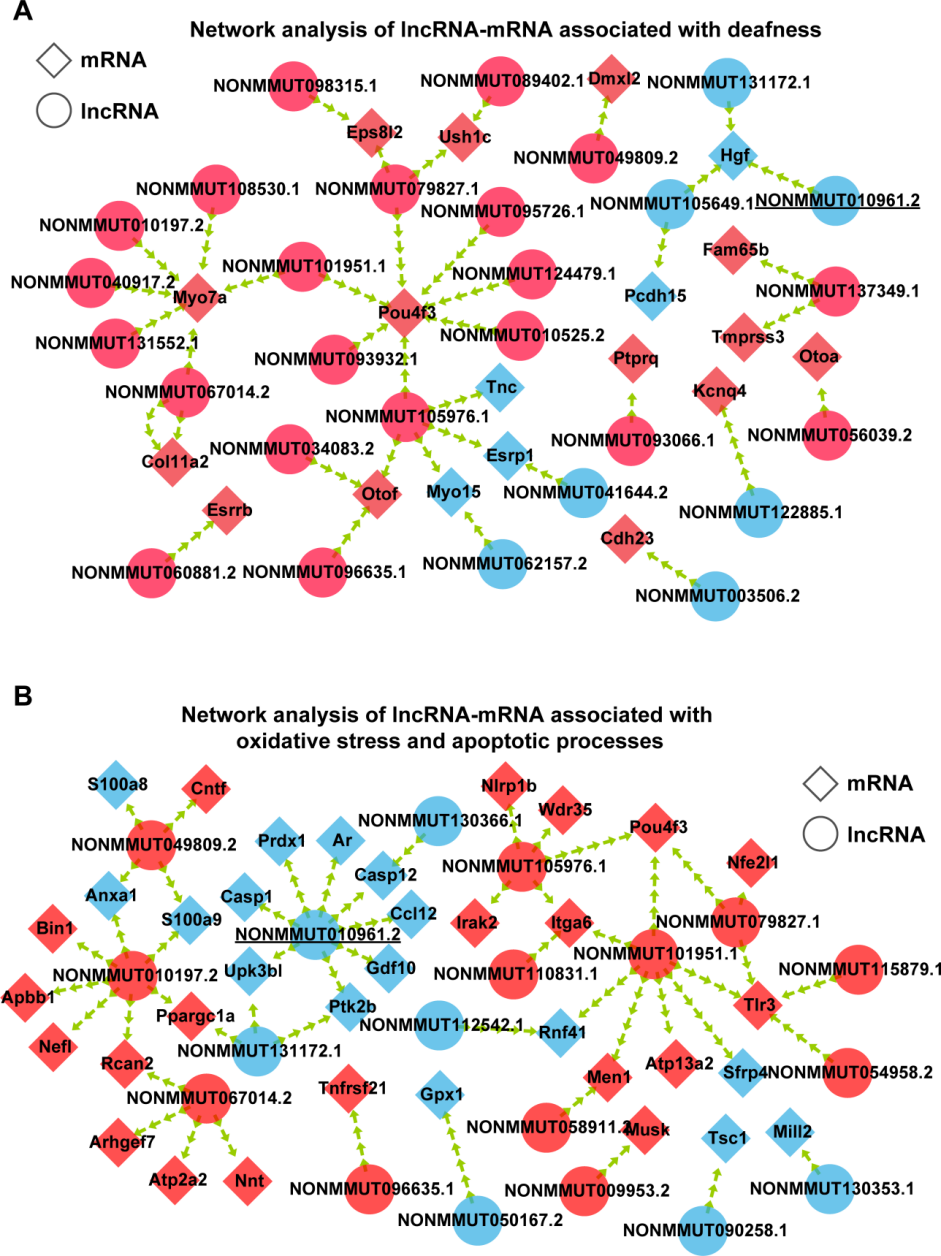
**Interaction of coexpressed mRNA-lncRNA.** (**A**) Network analysis of lncRNA-mRNA associated with deafness. (**B**) Network analysis of lncRNA-mRNA associated with oxidative stress and apoptotic processes. Circular nodes represent lncRNAs; diamond nodes represent mRNAs. Red nodes represent the downregulated transcripts, and blue nodes represent the upregulated transcripts.

To demonstrate which lncRNAs are involved in AHL, the interactions among the 34 hearing loss-associated mRNAs and coexpressed lncRNAs were first analyzed using Spearman correlation based on the RNA sequencing data. Two hundred seventy-nine lncRNAs were connected with the 34 mRNAs (Supplementary Figure 1A). Meanwhile, the interactions of coexpressed lncRNAs and 92 mRNAs associated with oxidative stress and apoptotic processes were analyzed. Four hundred seventy-three lncRNAs were coexpressed with the 92 mRNAs (Supplementary Figure 1B). We observed that some lncRNAs were shared by these two networks (Figure 5A, 5B). Finally, 11 lncRNAs of interest were selected to validate the reliability of the RNA-seq data (Table 1). Among these lncRNAs, two were significantly expressed but not included in these two networks. The 11 lncRNAs and their highest interacting and correlated target mRNAs in the two networks are shown in Table 2. These results suggest close regulation of mRNA expression by lncRNAs in hearing loss and oxidative stress during the development of AHL in C57BL/6J mice.

**Table 1:** Candidate lncRNAs for follow-up qRT-PCR validation.

**Unique ID**	**Regulation**	**Fold change**	**Relationship**
NONMMUT067014.2	down	0.29	sense_genic_nested_intronic
NONMMUT079827.1	down	inf	antisense_genic_nested_intronic
NONMMUT096635.1	down	0.49	antisense_intergenic_divergent_upstream
NONMMUT101951.1	down	0.32	antisense_genic_overlapping_exonic
NONMMUT105976.1	down	0.04	antisense_genic_nested_intronic
NONMMUT010961.2	up	5.88	antisense_genic_nested_intronic
NONMMUT131172.1	up	4.67	antisense_genic_overlapping_exonic
NONMMUT062631.2	up	2.89	antisense_genic_nested_exonic
NONMMUT021332.2	up	2.91	sense_genic_overlapping_exonic
NONMMUT010197.2	down	0.16	antisense_genic_overlapping_exonic
NONMMUT049809.2	down	0.23	sense_genic_nested_intronic

**Table 2:** Candidate lncRNA-mRNA pairs.

**Unique ID**	**mRNA**	**Gene name**	**Correlation**
NONMMUT067014.2	ENSMUST00000179939	Atp2a2	0.98
	ENSMUST00000107127	Myo7a	0.97
NONMMUT079827.1	ENSMUST00000167106	Tlr3	0.96
	ENSMUST00000140025	Eps8l2	0.97
NONMMUT096635.1	ENSMUST00000114747	Otof	0.96
	ENSMUST00000024708	Tnfrsf21	0.97
NONMMUT101951.1	ENSMUST00000025374	Pou4f3	0.97
	ENSMUST00000096386	Rnf41	0.98
NONMMUT105976.1	ENSMUST00000025374	Pou4f3	0.98
	ENSMUST00000025374	Pou4f3	0.98
NONMMUT010961.2	ENSMUST00000200189	Hgf	0.97
	ENSMUST00000052837	Ar	0.98
NONMMUT131172.1	ENSMUST00000200189	Hgf	0.96
	ENSMUST00000132734	Ppargc1a	-0.99
NONMMUT062631.2			
NONMMUT021332.2			
NONMMUT010197.2	ENSMUST00000107127	Myo7a	0.96
	ENSMUST00000117167	S100a9	-0.96
NONMMUT049809.2	ENSMUST00000144736	Dmxl2	0.96
	ENSMUST00000117167	S100a9	-0.97

### qRT-PCR validation of the expression levels of lncRNAs and their potential target mRNAs in animal and cellular models of AHL

**Figure 6:**
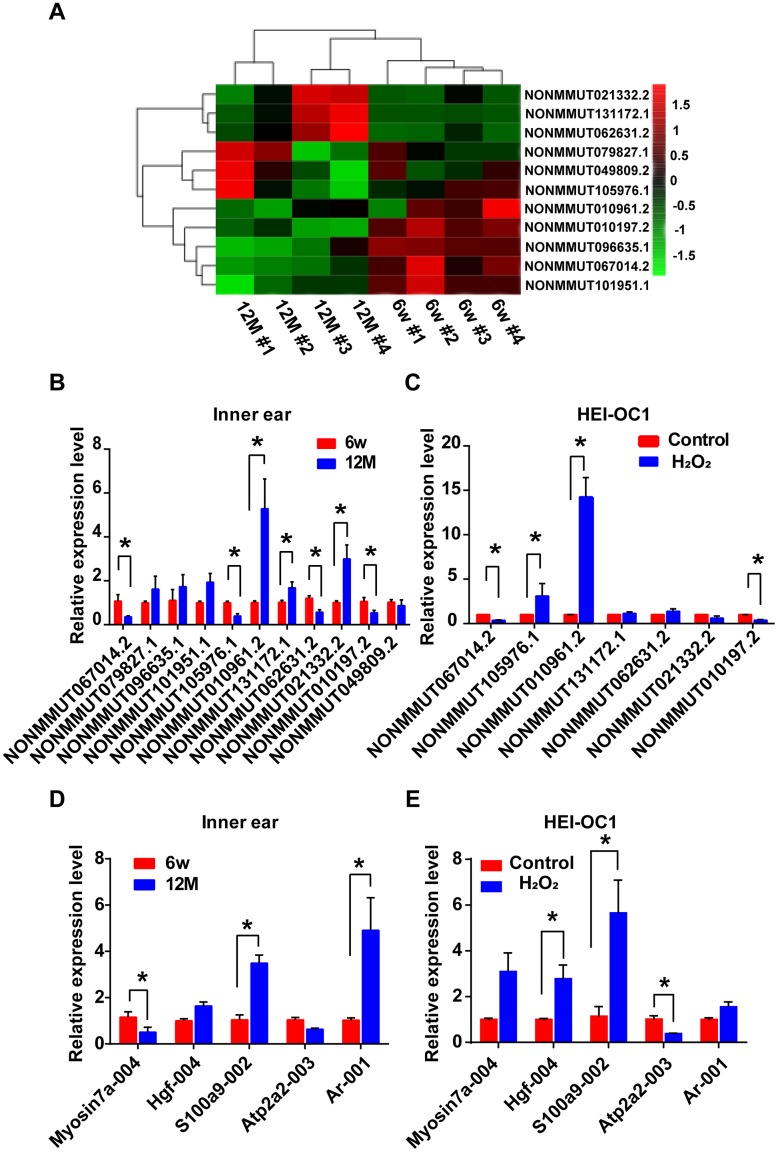
**Identification of differentially expressed lncRNAs and their potential target mRNAs in animal and cellular models of AHL.** (**A**) Heat map of candidate lncRNA expression levels in six-week-old and one-year-old mice. The gradient color barcode at the top right indicates the log_2_ FPKM. Relative expression of the selected lncRNAs (**B**) and mRNAs (**D**) in the cochlear tissues, as detected by qRT-PCR. Tissues from the 6w mouse group were used as a control. Relative expression of the selected lncRNAs (**C**) and mRNAs (**E**) in a cellular model of AHL. Cells not treated with H_2_O_2_ were used as a control. *P < 0.05 compared to control.

The analysis of hierarchical clustering of the 11 lncRNAs is shown in Figure 6A. To validate the RNA-sequencing results, we performed qRT-PCR analysis on the expression of the 11 candidate lncRNAs and their potential target mRNAs. Compared with the expression level of six-week-old mice, the expression of NONMMUT067014.2, NONMMUT105976.1, NONMMUT062631.2, and NONMMUT010197.2 was clearly decreased, while NONMMUT010961.2, NONMMUT131172.1, and NONMMUT021332.2 were significantly increased in one-year-old mice (*P <* 0.05). However, the difference in the expression levels of NONMMUT079827.1, NONMMUT096635.1, NONMMUT101951.1, and NONMMUT049809.2 between six-week-old and one-year-old mice was not statistically significant (Figure 6B). In the cellular model, seven differentially expressed lncRNAs in the animal model were chosen for qRT-PCR validation. Among these lncRNAs, the changes in the expression of NONMMUT067014.2, NONMMUT105976.1, NONMMUT010961.2, and NONMMUT010197.2 were statistically significant (Figure 6C). Meanwhile, some mRNAs that are highly correlated with NONMMUT067014.2, NONMMUT010961.2, and NONMMUT010197.2, including ATPase, Ca++ transporting, cardiac muscle, slow twitch 2 (*Atp2a2*), myosin VIIA (*Myo7a*), hepatocyte growth factor (*Hgf*), androgen receptor (*Ar*), and S100 calcium binding protein A9 (calgranulin B) (*S100a9*), were selected for further analysis using qRT-PCR in animal and cellular models of AHL (Table 2 and Figure 6D, 6E). In animal models, the differential expression of *Myo7a*, *S100a9* and *Ar* was significant (Figure 6D). In cellular models, the significantly differentially expressed mRNAs were *Hgf*, *S100a9* and *Atp2a2* (Figure 6E).

### Correlation of lncRNA NONMMUT010961.2 and its potential target mRNAs

**Figure 7:**
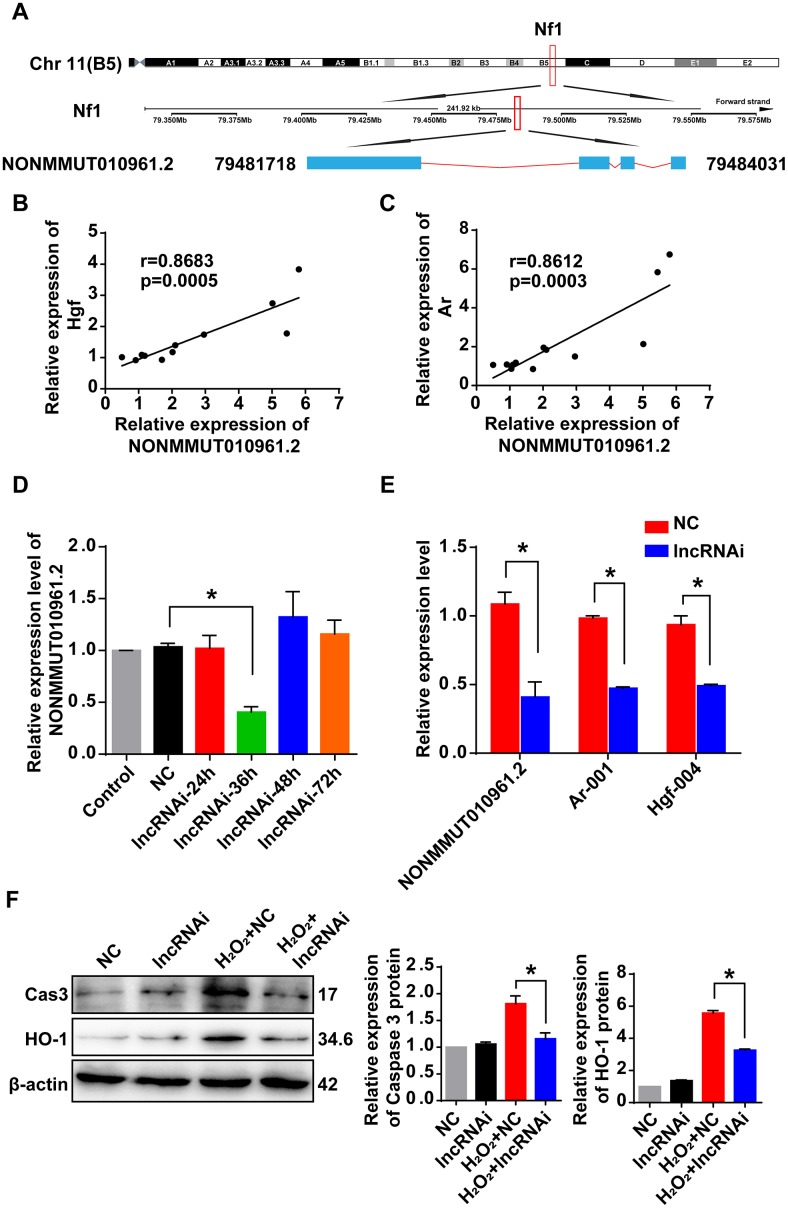
**Correlation of lncRNA NONMMUT010961.2 and its potential target mRNAs.** (**A**) The chromosome location of NONMMUT010961.2 in the mouse genome is shown. (**B**, **C**) Pearson’s correlation was used to analyze the positive correlation between lncRNA NONMMUT010961.2 expression and the expression level of *Hgf* and *Ar* in the 12 matched samples. (**D**) The relative expression level of lncRNA NONMMUT010961.2 in HEI-OC1 cells treated with lncRNAi for 24 h, 36 h, 48 h and 72 h compared with those treated with NC. *P < 0.05 compared to NC. (**E**) qRT-PCR analysis showed that lncRNA NONMMUT010961.2 and its potential target mRNAs *Hgf* and *Ar* decreased in HEI-OC1 cells treated with lncRNAi for 36 h compared with those treated with NC. *P < 0.05 compared to NC. (**F**) Western blot analysis showed that cleaved caspase 3 and HO-1 were decreased by lncRNAi in the AHL cellular model. (*P < 0.05).

The lncRNA NONMMUT010961.2 was chosen as an example to study the correlation pattern among lncRNA and its potential target mRNAs, because it showed the largest changes in expression. The sequence of this lncRNA was first analyzed using the open reading frame finder from the National Center for Biotechnology Information (NCBI). LncRNA NONMMUT010961.2 was located on chromosome 11 in the genome, and its transcription site was mapped in the intron of its host gene encoding recombinant neurofibromin 1 (*Nf1*) from 79,481,718 to 79,484,031 (Figure 7A). Then, we analyzed the correlation of lncRNA NONMMUT010961.2 and its potential target mRNAs by Pearson’s correlation. The expression levels of *Hgf* and *Ar* were positively correlated with that of NONMMUT010961.2 (Figure 7B, 7C), which indicates that NONMMUT010961.2 is associated with oxidative stress and AHL. To further validate the correlation of lncRNA NONMMUT010961.2 and its potential target mRNAs *Hgf* and *Ar*, a smart silencer targeting lncRNA NONMMUT010961.2 (lncRNAi) was designed in this study. First, HEI-OC1 cells were treated with lncRNAi for 24 h, 36 h, 48 h and 72 h to determine the optimal transfection time (Figure 7D). qRT-PCR analysis showed that lncRNA NONMMUT010961.2 was decreased 36 h after lncRNAi treatment. Therefore, we used 36 h for the transfection time of other experiments in HEI-OC1 cells. Next, we knocked down lncRNA NONMMUT010961.2 using lncRNAi to detect whether the expression of its target mRNAs was affected. As expected, *Hgf* and *Ar* were downregulated in lncRNA NONMMUT010961.2 knockdown cells compared with NC cells (Figure 7E). Western blotting results showed that the expression levels of cleaved caspase 3 and HO-1 were decreased by lncRNAi in the AHL cellular model (Figure 7F). Taken together, these results suggest that the lncRNA NONMMUT010961.2 stimulates its potential target mRNAs in HEI-OC1 cells; therefore, lncRNA NONMMUT010961.2 induces oxidative stress and AHL development in HEI-OC1 cells.

## DISCUSSION

Age-related hearing loss (AHL) is a complex degenerative disease that gradually results in a heavy economic burden for society [2]. The C57BL/6J mouse strain is one of the most widely used models to study age-associated diseases and was chosen as an AHL animal model for RNA-seq analysis in this study. In this study, cochleae of six-week-old and one-year-old C57BL/6J mice were first used to characterize the expression profile of lncRNAs and mRNAs using RNA-seq. Then, we further investigated the potential role of lncRNA NONMMUT010961.2 in the pathogenesis of AHL using coexpressed mRNAs that are associated with hearing loss, oxidative stress and apoptotic processes.

Oxidative stress plays a key role in the development of AHL [19, 23]. Reactive oxygen species (ROS) are primarily produced from mitochondria. The accumulation of ROS could impair antioxidant functions and damage macromolecules, such as nuclear DNA, mitochondrial DNA (mtDNA), membranes, and proteins. Moreover, accumulation of macromolecule mutations is a causative factor for apoptosis of hair cells and thus the development of AHL [2, 23]. Hydrogen peroxide (H_2_O_2_) is produced by diverse cellular pathways and decomposed into water (H_2_O) and oxygen by catalase and glutathione peroxidase (Gpx). Once the balance of intracellular concentrations of H_2_O_2_ is broken, oxidative stress will be caused [24]. An increasing number of studies note that cultured cells are exposed to H_2_O_2_ as experimental models of oxidative stress [25, 26], and there have been such studies in HEI-OC1 cells [9, 21, 22]. Meanwhile, GO-BP analysis revealed some significant differentially expressed mRNAs enriched in oxidative stress and apoptotic processes in RNA-seq (Figure 4A). Thus, HEI-OC1 cells exposed to 920 μM H_2_O_2_ were used as the cellular model of AHL in our study.

GO terms are grouped into three ontologies: biological processes, cellular components, and molecular functions. Among the top 20 biological processes, 29 mRNAs were associated with sensory perception of sound (GO: 0007605). Meanwhile, we identified 34 hearing loss-related mRNAs that were significantly differentially expressed between the cochleae of six-week-old and one-year-old C57BL/6J mice. Interestingly, the 34 mRNAs included all 29 mRNAs enriched in GO: 0007605. These 34 mRNAs included the genes that participated in the shaping of the hair bundle, such as Usher syndrome type 1C (*Ush1c*), *Myo7a*, protocadherin 15 (*Pcdh15*) [27], Eps8-related protein 2 (*Eps8l2*) [28] protein-tyrosine phosphatase, receptor-type, q (*Ptprq*) [29] and cadherin 23 (*Cdh23*) [30]; the genes associated with hair cell function, such as Pou domain, class 4, transfection factor 3 (*Pou4f3*) [31, 32], potassium channel, voltage-gated, kqt-like subfamily, member 4 (*Kcnq4*) [33, 34], diaphanous-related formin 1 (*Diaph1*) [35]and lipoxygenase homology domain-containing 1 (*Loxhd1*) [36]; the genes related to hair cell stereocilia, such as myosin XVA (*Myo15a*) [37] and family with sequence similarity 65, member b (*Fam65b*) [38]; the genes involved in the tectorial membrane, such as carcinoembryonic antigen-related cell adhesion molecule 16 (*Ceacam16*) [39] and tectorin alpha (*Tecta*) [40]; and other genes essential for inner ear development and function, such as *Hgf* [41], direct iap-binding protein with low pi (*Diablo*) [42], estrogen-related receptor beta (*Esrrb*) [43], otoancorin (*Otoa*) [44], otogelin-like protein (*Otogl*) [45] and dmx-like 2 (*Dmxl2*) [46]. These results suggest that the differentially expressed lncRNAs identified by this study most likely play roles in AHL by regulating the expression of the above mentioned genes.

In the present study, we selected 11 candidate lncRNAs associated with AHL from the two mRNA-lncRNA coexpression networks (Supplementary Figure 1). According to qRT-PCR analysis, the significantly differentially expressed lncRNA NONMMUT010961.2 in both animal and cellular models was selected to study its relationship with its potential mRNA targets. The most relevant mRNAs with lncRNA NONMMUT010961.2 are *Hgf* and *Ar* in the two networks.

*Hgf* maps to mouse chromosome 5. This gene encodes a protein that binds to the hepatocyte growth factor receptor to regulate cell growth, cell motility and morphogenesis in numerous cell and tissue types. Schultz et al. [41] generated mice with a conditional knockout of *Hgf* in the inner ear and observed disorganized tectorial membrane, thin and flattened stria vascularis with occasional clumps of cellular proliferation, hypoplastic spiral ganglion, and OHC degeneration throughout the organ of Corti. Meanwhile, MH19-*Hgf* transgenic mice overexpressing *Hgf* had ABR thresholds 60-dB greater than those of the littermate controls on average. These results demonstrated that the *Hgf* gene is essential for cochlear function. In this study, we found that *Hgf* is upregulated in one-year-old mice compared with six-week-old mice, and there is a strong coexpression relationship between lncRNA NONMMUT010961.2 and *Hgf* (Figure 7E)*.* Therefore, we hypothesize that lncRNA NONMMUT010961.2 induces the development of AHL by stimulating *Hgf* expression. *Ar* is located at chromosome X in the mouse genome. The encoded protein is a key regulator of signaling by androgens, a class of steroid hormones involved in male reproductive development. Feng et al. [47] noted that ROS activate *Ar* functions by the TXNDC9-PRDX1 axis in prostate cancer (PCa) cells. In the present study, we observed the highest correlation between lncRNA NONMMUT010961.2 and *Ar* (Supplementary Figure 1B and Table 2). Thus, it is likely that lncRNA NONMMUT010961.2 participates in AHL by regulating Ar expression.

Many lncRNAs leave the site of transfection and function in *trans*. For example, Rinn et al. [48] found that lncRNA HOTAIR is required to maintain a transfectionally silent chromosomal mark in *trans* on the distant *HOXD* locus. LincRNA-EPS (lincRNA erythroid prosurvival) repressed apoptosis by suppressing the expression of the pro-apoptotic gene *Pycard* in *trans* [49]. In this study, lncRNA NONMMUT010961.2 acted on its target mRNAs *Hgf* and *Ar* in *trans*-regulation. Clearly, experiments will be designed in the future to determine this pattern of regulation in *trans*.

In summary, our results demonstrate that lncRNAs and mRNAs undergo changes in expression during the development of AHL. LncRNA NONMMUT010961.2 was determined to participate in AHL by stimulating *Hgf* and *Ar* in *trans*-regulation. These findings may provide a novel direction for developing effective treatments for AHL. Further studies are needed to validate the ceRNA mechanisms of these candidate lncRNAs and mRNAs in relation to AHL.

## MATERIALS AND METHODS

### Animals

C57BL/6J mice (34 male and 36 female) were used in our experiments. The mice were divided into two groups: the six-week-old mice group with ages of 1–1.5 months (16 male and 16 female) and the one-year-old mice group with ages of 12–15 months (18 male and 20 female). The procedures involving the use and care of the animals were reviewed and approved by the Animal Center of Binzhou Medical University.

### RNA extraction

Samples (four pairs of cochleae of the six-week-old mice group and four pairs of cochleae of the one-year-old mice group) were used for lncRNA and mRNA expression analysis. High-throughput RNA sequencing was performed by Annoroad Gene Tech Co, Ltd. (Beijing, China). Total RNA was extracted using TRIzol Reagent (Invitrogen, Carlsbad, CA, USA) according to the manufacturer’s protocols. RNA purity was checked using the KaiaoK5500® Spectrophotometer (Kaiao, Beijing, China). RNA integrity and concentration were assessed using the RNA Nano 6000 Assay Kit of the Bioanalyzer 2100 system (Agilent Technologies, CA, USA). rRNA was removed using Epicenter Ribo-Zero^TM^ Gold Kits (Human/Mouse/Rat/other) (Epicenter, USA).

### Analysis of RNA-sequencing data

Analysis of RNA-sequencing data was performed by Yunlong Zhang affiliated with Annoroad Gene Tech Co, Ltd. (Beijing, China). To ensure the quality of the data, raw data were processed with Perl scripts. Reads in the following category were filtered out: 1) Adapter-polluted reads. (Reads containing more than 5 adapter-polluted bases were regarded as adaptor-polluted reads and were filtered out.) 2) Low-quality reads. (Reads with the number of bases whose phred quality value was no more than 19 accounting for more than 15% were regarded as low-quality reads and were filtered out.) 3) Reads with more than 5% N bases accounting. Clean data were mapped to the reference genome using HISAT2 (http://ccb.jhu.edu/software/hisat2/index.shtml). The reference genomes and the annotation file were downloaded from the ENSEMBL database (http://www.ensembl.org/index.html).

HTSeq (http://www-huber.embl.de/users/anders/HTSeq/doc/overview.html) was used for read count of each gene in each sample, and FPKM (Fragments Per Kilobase Million Mapped Reads) was then calculated to represent the expression level of genes in each sample. The formula was defined as FPKM = 10^6^×F/ (NL×10^-3^), where F is the number of fragments assigned to a certain gene in a certain sample, N is the total number of mapped reads in the certain sample, and L is the length of the certain gene. Thus, RPKM eliminates the influences of different transcript lengths and sequencing discrepancies on the calculation of expression.

DEGseq (http://www.bioconductor.org/packages/release/bioc/html/DEGseq.html) or DESeq (http://www.bioconductor.org/packages/release/bioc/html/DESeq.html) were used for differential expression analysis of two samples with or without replicates. A *P*-value could be assigned to each gene and adjusted by BH. Genes with *q* <0.05 and |log_2_ ratio| >1 were identified as differentially expressed genes.

### GO and KEGG pathway enrichment analysis

GO (Gene Ontology, http://geneontology.org/) enrichment analysis was performed for all differentially expressed mRNAs, and a hypergeometric *p*-value was calculated and adjusted as the *q*-value. GO terms with *q*<0.05 were considered to be significantly enriched. GO enrichment analysis showed the biological functions of differentially expressed mRNAs. KEGG (Kyoto Encyclopedia of Genes and Genomes, http://www.kegg.jp/) is a database resource that contains a collection of manually drawn pathway maps representing our knowledge of the molecular interaction and reaction networks. Using the same method as GO enrichment analysis, significantly enriched KEGG pathways were identified.

### mRNA-lncRNA coexpression network

The mRNAs with high Spearman correlation coefficients (*p* > 0.9) were selected as the *trans*-targets. In addition, mRNAs with distances less than 50 kb were selected as *cis*-targets. The mRNA-lncRNA coexpression network was generated by Cytoscape software (v3.2.1).

### Auditory brainstem response (ABR)

ABR is a common method to assess the function of the cochlea. Mice were anesthetized by intraperitoneal injections of chloral hydrate (0.48 mg/kg). During testing, mice were always placed in a soundproof chamber. The acoustic signals of click and 8-, 16-, or 32 kHz pure tone bursts were generated and channeled into the animals’ ear canals. The amplified brainstem responses were recorded and processed by SmartEP (Intelligent Hearing Systems, USA) through three subdermal electrode needles: the active electrode was inserted at the vertex, the reference electrode was inserted under the test ear, and the ground electrode was inserted under the contralateral ear. The stimulus intensities were delivered in 10 dB descending steps and 5 dB increasing steps to identify the lowest stimulation decibel level at which detectable responses were elicited.

### Quantitative real-time polymerase chain reaction (qRT-PCR)

The dissected cochleae were stored at -80 °C. Total RNA was extracted from frozen cochlear tissues or HEI-OC1 cells using TRIzol Reagent (Invitrogen, USA) following the manufacturer’s protocol. RNA quality and concentration were measured by a NanoDrop 2000c spectrophotometer (Thermo Scientific, USA). Genomic DNA was removed, and cDNA was synthesized using the PrimeScript^TM^ RT reagent kit (TaKaRa, China). Real-time PCR was performed with SYBR Green Master (Roche, Switzerland) using a multicolor real-time PCR detection system (Bio-Rad iQ5, USA). PCR analyses were performed using the following parameters: 95 °C for 10 min followed by 40 cycles at 95 °C for 15 s and 60 °C for 30 s. The relative RNA amounts were calculated by a 2^-ΔΔCT^ method. Glyceraldehyde-3-phosphate dehydrogenase (GAPDH) was used as an internal control for the relative quantitation of lncRNAs. All primers used in the RT-PCR are shown in Table 3, which were designed and synthesized in RiboBio (Guangzhou, China) and TaKaRa (Dalian, China).

**Table 3:** Sequences of primers used for qRT-PCR.

**Unique ID**	**Forward primer**	**Reserve primer**
NONMMUT067014.2	5’-agacattcctaccagtgcttcca-3’	5’-ctccactgttctgctcggtct-3’
NONMMUT079827.1	5’-tcttctccctccagttcctca-3’	5’-actcacatttgccctcctgtct-3’
NONMMUT096635.1	5’-cactgtagaaggcacagcagaga-3’	5’-atttggtggatgagaagggaga-3’
NONMMUT101951.1	5’-ctaaccctagccttgaactcacaga-3	5’-acacaaaggaaggaagaaaggatg-3’
NONMMUT105976.1	5’-actcaggagaggcatccacac-3’	5’-cagtcccaggctaaaacgatg-3’
NONMMUT010961.2	5’-gaactcggagagtgagaagagactt-3’	5’-gcttggtcatcgttttcttttg-3’
NONMMUT131172.1	5’-gctctgtcgctgtctgcttt-3’	5’-cggtgatggaatgtgatgct-3’
NONMMUT062631.2	5’-ctcacttacatgcaaggacttgct-3’	5’-gtcccatggtctttacttcttcatc-3’
NONMMUT021332.2	5’-agtcagattccaagcaatagaacag-3’	5’-tcttttgggtaagatcaagtgcag-3’
NONMMUT010197.2	5’-tccagctgtctgcaaatatcactc-3’	5’-gctagtgttgctcttcgtctcca-3’
NONMMUT049809.2	5’-gtgactcatacctgcctgtaactcc-3’	5’-ggcgtgctctaggtgctcaat-3’
Atp2a2-003	5’-ctgttctgccgcatagtaggat-3’	5’-accaacctacatacaacgggc-3’
Ar-001	5’-ggattctgtgcagcctattgc-3’	5’-tcaggaaagtccacgctcac-3’
S100a9-002	5’-ggccaacaaagcaccttctc-3’	5’-ttctctttcttcataaaggttgcca-3’
Myo7a-004	5’-ttcagcaactacagccaggg-3’	5’-ccagggaggacaccttatcg-3’
Hgf-004	5’-tgatcccccatgaacacagg-3’	5’-tagagaacaagtgcgtgcct-3’
GAPDH	5’-aggtcggtgtgaacggatttg-3’	5’-tgtagaccatgtagttgaggtca-3’

### HEI-OC1 cell culture

The House Ear Institute-Organ of Corti 1 (HEI-OC1) cells were cultured in high glucose Dulbecco’s Modified Eagle Medium (DMEM, Gibco, #11965092) containing 10% fetal bovine serum (FBS, Gibco, #10437028) without antibiotics at 33 °C and 10% CO_2_.

### Western blot analysis

HEI-OC1 cells cultured in 6-cm plates were washed with ice-cold PBS and then collected by cell scrapers in microcentrifuge tubes and centrifuged at 2,000 rpm for 10 min at 4 °C. Cells were lysed with 70 μl of RIPA buffer (Thermo Scientific, USA), including a protease and phosphatase inhibitor cocktail (Roche, Switzerland), for 30 min on ice. After centrifugation at 13,000 × g for 30 min at 4 °C, supernatants were collected, and the protein concentration was quantified using BCA assays (Takara, Japan). A loading buffer was added to equal amounts (30 μg) of protein for each sample and boiled for 5 min to denature the proteins. The samples were then separated by SDS-PAGE on a 10% gel (Beyotime, China) and electrotransferred to PVDF membranes (Invitrogen, USA). The membranes were blocked with 5% nonfat dried milk in Tris buffered saline with 0.1% Tween 20 (TBS-T) for 2 h at room temperature. The membranes were incubated with the following primary antibodies: anti-Caspase 3 (1:1000, Novous, NB100-56708), anti-HO-1 (1:1000, Abcam, ab13248), and anti-β-actin (1:1000, Proteintech, 20536-1-AP) overnight at 4 °C. After washing with TBST, membranes were incubated for 1 h at room temperature with a secondary antibody followed by three 10-min washes with TBST. Protein bands were visualized using an enhanced chemiluminescence kit (Bio-Rad, USA). The relative densities of the protein bands were determined using an image acquisition and analysis system (Bio-Rad, USA). β-actin was used to normalize protein loading.

### CCK-8 cell viability assay

Cell viability was detected by a CCK-8 (Cell Counting Kit-8) assay kit (Dojindo, Japan). HEI-OC1 cells were seeded in each well of a 96-well plate with the concentration adjusted to 1.3 × 10^4^ cells per well and incubated overnight at 33 °C and 10% CO_2_. The next day, the cells were treated with H_2_O_2_. After 24 h of treatment at 33 °C, a CCK-8 assay was performed following the manufacturer’s protocol. Absorbance at 450 nm was measured using a microplate reader (BioTek, USA).

### Real-time cell analyzer

Cytotoxicity was monitored by the xCELLigence Real-Time Cell Analyzer (RTCA) DP system (ACEA Biosciences, USA) as described [50]. First, the background of the E-plates was determined in 50 μl of medium, and subsequently, 100 μl of the HEI-OC1 cell suspension was added (1.3 × 10^4^ cells per well). Cells were incubated for 30 min at room temperature, and E-plates were placed into the RTCA station. Cells were grown for at least 24 h, with impedance being measured every 15 min. After the designated treatments, cells were monitored again every 15 min until the end of the experiment. The electronic readout, cell-sensor impedance induced by adherent cells to the electron flow, is displayed as an arbitrary unit, known as the cell index. The normalized cell index was calculated by the RTCA software at the selected normalization time point, which was chosen as the time immediately before the addition of treated drugs. Each treatment was performed in triplicate.

### Histological analyses of inner ears

Six-week-old and one-year-old C57BL/6J mice were sacrificed (n = 3 per group), and their inner ears were dissected and fixed with 4% paraformaldehyde for 24 h and then decalcified in 10% EDTA for 4–5 days. After decalcification, the inner ears were embedded in paraffin. The tissue sections (5 μm) were mounted on glass sides, stained in hematoxylin and eosin (H&E), and finally examined under a light microscope (Leica, Germany).

### RNA interference

A smart silencer targeting lncRNA NONMMUT010961.2 (lncRNAi) and its negative control (NC) were synthesized by RiboBio (Guangzhou, China). The lncRNAi target sites were “5’-GATGAAGCTGTTACTACAA-3’, 5’-GGAACAATTTCAACAACAA-3’, 5’-TAACAAATACCACCTCAAA-3’, 5’-AGCAAAAGAAAACGATGACC-3’, 5’-CTACCACCTCAAATTCTACA-3’, and 5’-CACAGGCCATATCAAGACCA-3’. Cells were transfected with 100 nM lncRNAi using the riboFECT^TM^ CP Transfection Kit (Guangzhou, China) in accordance with the manufacturer’s instructions. HEI-OC1 cells were plated into six-well plates and transfected with lncRNAi and NC. The expression level of lncRNA NONMMUT010961.2 was detected by qRT-PCR.

### Statistical analysis

Data are presented as the mean ± SEM, and statistical analyses were performed using ANOVA or unpaired Student’s t-test with GraphPad Prism 6. *P* < 0.05 was considered to be significant.

## Supplementary Material

Supplementary Figure 1
