# Supplementary material for: RNA-seq analysis of potential lncRNAs for age-related hearing loss in a mouse model
Source: Aging (Albany NY). 2020 Apr 26;12(8):7491–510. doi: 10.18632/aging.103103 (PMC7202524; doi:10.18632/aging.103103)
Supplement: Supplementary Figure 1 [file aging-12-103103-s001..pdf]

SUPPLEMENTARY FIGURE

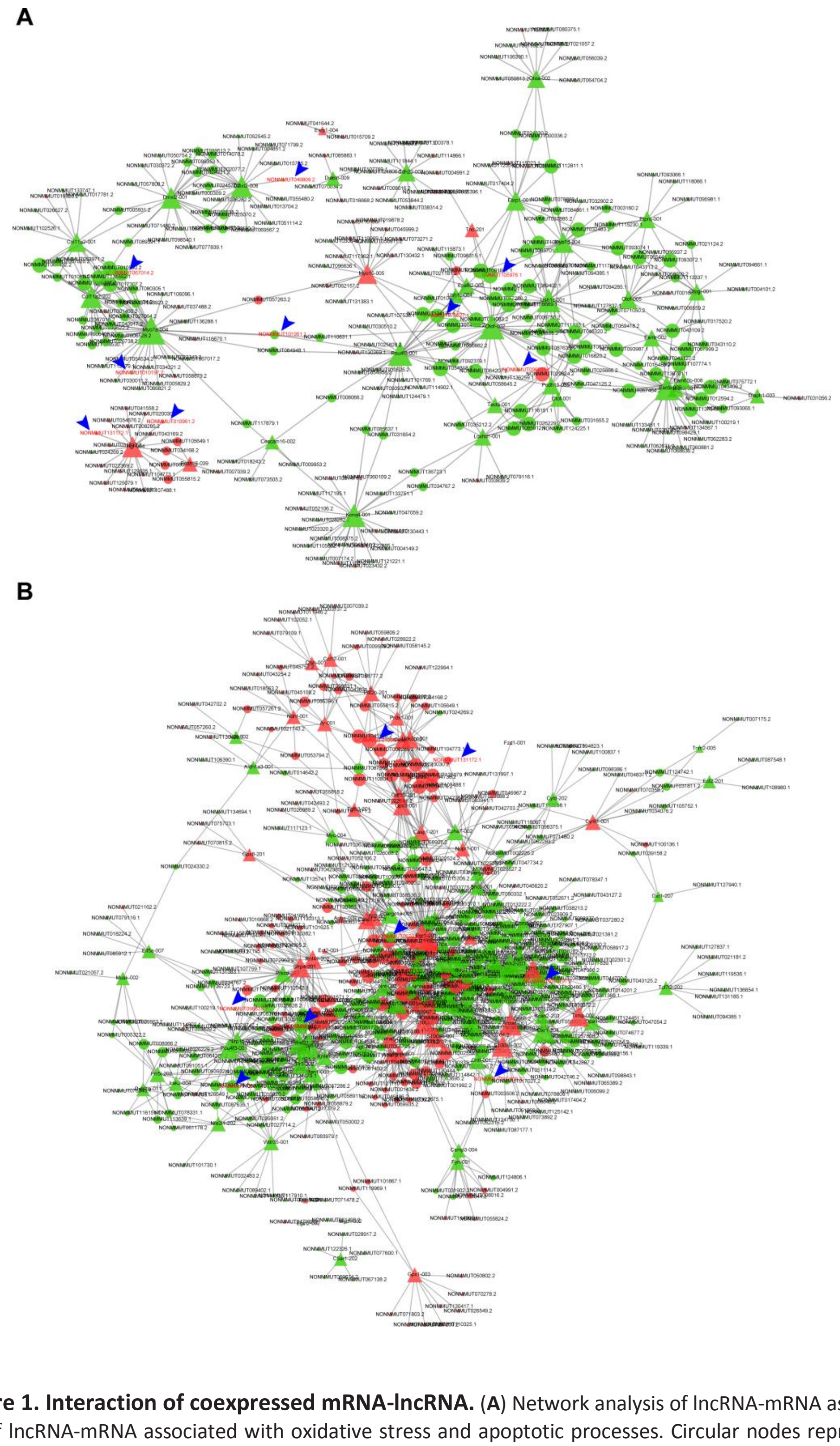

Supplementary Figure 1. Interaction of coexpressed mRNA-lncRNA. (**A**) Network analysis of lncRNA-mRNA associated with deafness. (**B**) Network analysis of lncRNA-mRNA associated with oxidative stress and apoptotic processes. Circular nodes represent lncRNAs; triangle nodes represent mRNAs. Red nodes represent the upregulated transcripts, and green nodes represent the downregulated transcripts. A greater node of mRNAs represents more lncRNAs acting on this one, and a greater node of lncRNAs means more mRNAs are acting on by this one. The selected lncRNAs are marked by the blue arrow.
